# Implementation of the Observational Medical Outcomes Partnership Model in Electronic Medical Record Systems: Evaluation Study Using Factor Analysis and Decision-Making Trial and Evaluation Laboratory-Best-Worst Methods

**DOI:** 10.2196/58498

**Published:** 2024-09-27

**Authors:** Ming Luo, Yu Gu, Feilong Zhou, Shaohong Chen

**Affiliations:** 1 Meizhou People's Hospital Meizhou China; 2 Shenzhen Luohu District People's Hospital Shenzhen China

**Keywords:** electronic medical record, Technology Acceptance Model, external factors, perception, attitude, behavioral inclination, OMOP

## Abstract

**Background:**

Electronic medical record (EMR) systems are essential in health care for collecting and storing patient medical data. They provide critical information to doctors and caregivers, facilitating improved decision-making and patient care. Despite their significance, optimizing EMR systems is crucial for enhancing health care quality. Implementing the Observational Medical Outcomes Partnership (OMOP) shared data model represents a promising approach to improve EMR performance and overall health care outcomes.

**Objective:**

This study aims to evaluate the effects of implementing the OMOP shared data model in EMR systems and to assess its impact on enhancing health care quality.

**Methods:**

In this study, 3 distinct methodologies are used to explore various aspects of health care information systems. First, factor analysis is utilized to investigate the correlations between EMR systems and attitudes toward OMOP. Second, the best-worst method (BWM) is applied to determine the weights of criteria and subcriteria. Lastly, the decision-making trial and evaluation laboratory technique is used to illustrate the interactions and interdependencies among the identified criteria.

**Results:**

In this research, we evaluated the AliHealth EMR system by surveying 98 users and practitioners to assess its effectiveness and user satisfaction. The study reveals that among all components, “EMR resolution” holds the highest importance with a weight of 0.31007783, highlighting its significant role in the evaluation. Conversely, “EMR ease of use” has the lowest weight of 0.1860467, indicating that stakeholders prioritize the resolution aspect over ease of use in their assessment of EMR systems.

**Conclusions:**

The findings highlight that stakeholders prioritize certain aspects of EMR systems, with “EMR resolution” being the most valued component.

## Introduction

### Background

Today, electronic files are a standard tool for enhancing the efficiency and effectiveness of care services, thanks to their speed, accuracy, intelligent systems, reminders, and decision support. One of the most commonly used systems is the electronic medical record (EMR), a computerized system used by care providers, including hospitals and doctors’ offices [1,2]. It is designed for entering, storing, displaying, retrieving, and printing patients’ medical records. The system offers several benefits, such as enhancing the quality of care provided to patients, better organizing information, and improving the timeliness, accuracy, and completeness of documentation [3]. Several factors drive health care providers and organizations to adopt EMR systems now or in the near future [4-6]. These include the ability to create and access patient records electronically, allowing patients to access their own files, preventing medication errors and allergic reactions, reducing medical errors, and providing immediate access to information across different locations. Additionally, EMR systems offer decision-support technology, streamline workflows, and enhance the overall efficiency of clinical processes. They also improve the quality of treatment and facilitate information sharing between general practitioners and specialists. Reducing medical errors and improving clinical data collection are key benefits of EMR systems. Health care providers and organizations increasingly recognize the need to adopt EMR systems to deliver more effective and efficient services [7]. However, the implementation of EMRs faces resistance, particularly from health care personnel, including doctors. To address this challenge, it is essential to create the necessary conditions for the acceptance of this system [8].

The acceptance of an EMR system is based on 2 major factors: perceived usefulness and perceived ease of use [9]. An individual’s perception of the usefulness of information technology refers to the belief that using a particular technology will improve job performance or facilitate more efficient task execution within the organization [10]. This support may be reflected in reduced task completion time or the provision of timely information [11,12]. Perceived ease of use reflects the collective belief within an organization that a specific system is straightforward and requires minimal effort to operate [13-16]. In essence, tasks that are perceived as simpler are more likely to be embraced by users. The primary aim of this research is to explore the factors influencing the acceptance and use of the EMR system and the Observational Medical Outcomes Partnership (OMOP) using the Technology Acceptance Model. According to the Davis model [17-20], these factors include external variables (eg, user interface design, data quality, and health information within the EMR system), perceived usefulness, perceived ease of use, attitudes toward the system, and the behavioral intention to use the EMR system. In China, several EMR systems are widely used, driven by significant government investments and initiatives to enhance health care information technology infrastructure. Notable systems include WeDoctor (WeDoctor Holdings Co Ltd), AliHealth (Alibaba Health/Alibaba Group), Ping An Good Doctor (Ping An Healthcare and Technology), Winning Health Technology Group, and Neusoft Corporation. WeDoctor and AliHealth dominate the market by offering comprehensive services with a strong focus on interoperability and data integration. Ping An Good Doctor integrates its EMR system with its online health platform, facilitating remote consultations. Winning Health Technology Group and Neusoft Corporation focus on hospital management and clinical information systems, offering specialized solutions for various health care settings. We herein examined the AliHealth EMR system and surveyed 98 users and practitioners to evaluate its effectiveness and user satisfaction.

In this study, 3 distinct methodologies are used to explore various aspects of health care information systems. First, factor analysis (FA) is used to investigate the correlation between EMR systems and attitudes toward the OMOP. This approach aims to uncover underlying relationships and dependencies between these 2 key elements. Second, the best-worst method (BWM) is applied to determine the weight of criteria and subcriteria, offering a structured and quantitative assessment of their significance. Finally, the decision-making trial and evaluation laboratory (DEMATEL) method is applied to illustrate the interactions and interdependencies among the identified criteria, shedding light on the complex relationships within the health care information system landscape. Together, these methodologies provide a comprehensive understanding of the complex dynamics and factors influencing EMRs and health care information management. This approach differs from earlier studies in several ways. First, it significantly reduces the number of survey questions required to create a systematic causality diagram that decision makers can use. In the context of enhancing health care quality through the implementation of the OMOP shared data model in EMR systems, the integration of the BWM and DEMATEL is crucial for a thorough analysis. The BWM is first used to determine the relative importance of various criteria by comparing the best and worst criteria against all others, generating a weighted set of criteria. These weights are then used as inputs for the DEMATEL method, which maps out the cause-and-effect relationships among the criteria. Specifically, the numerical results from BWM prioritize the importance of factors, and DEMATEL quantifies the degree of influence each factor has over others, thereby creating a structured network of interdependencies. This integration allows for a nuanced understanding by using BWM-derived weights to adjust the influence degrees determined by DEMATEL, resulting in a refined model that better predicts and enhances health care outcomes through the OMOP model. The research contribution is summarized in 3 main stages:

- Determining the correlation between EMR systems and attitudes toward OMOP using FA.
- Establishing the weight of criteria and subcriteria using the BWM.
- Illustrating the interactions and dependencies among the criteria using the DEMATEL method.

The subsequent sections of this document are organized as follows: The “Literature Review” provides a concise summary of the current literature on EMRs, with a specific focus on the OMOP. The “Methods” section details the research methodology used in this study, including a comprehensive description of the chosen strategies and methodologies. The “Results” section presents demographic information and summarizes the conclusions of the study. The “Discussion” section examines the findings of the research. The “Conclusions” section presents the final outcomes of the article.

### Literature Review

One of the most discussed aspects of eHealth today is EMRs. EMRs form the foundation of eHealth applications by storing patients’ medical histories. They also include legal documents created in both in-house and outpatient settings [21]. The electronic health record (EHR) system relies on these files for its data. Despite the widespread use of EMR systems in hospitals, many in the medical field still lack confidence in them [22]. Research on EMR systems in hospital settings remains limited. To address the personal, privacy, and security aspects affecting EMR adoption and utilization, Enaizan et al [23] introduced a decision support review framework. This framework is based on a multicriteria approach and K-means clustering, derived from insights gathered from Malaysian health care professionals. Although EHRs represent a significant technological advancement for health care, their adoption has been slow. Liou et al [24] highlighted this issue and proposed a theoretical framework to investigate and improve EHR utilization. Their framework uses the Technology-Organization-Environment model and the DEMATEL approach to create an Influence Network Relationship Map. This map integrates the core concepts of the Analytic Network Process with a modified Vlsekriterijumska Optimizacija i Kompromisno Resenje (VIKOR) method. This approach helps us better understand and utilize EHR technology. Oja et al [10] presented the process of converting EHR, claims, and prescription data into the OMOP format. They detailed the challenges and solutions associated with this conversion process.

EMRs are computerized medical information systems that capture, store, and display patient information. They assist doctors in conducting better, safer, and more efficient work, ultimately improving patient well-being. However, their adoption is still limited globally. Therefore, management information system scholars should investigate the increasing use of EMRs in the health care sector. Despite the potential of EMR systems to reduce administrative costs and medical errors, their adoption rates in physician practices have been slow. To address this, Zaidan et al [25] conducted a comparative study using multicriteria decision-making (MCDM) methods to assess and select open-source EMR software. The medical field is undergoing unprecedented transformation in many countries. Health care organizations are leveraging EMRs to enhance technology utilization, decision-making, and the search for medical solutions. There are employment opportunities for health care workers involved in the transition from paper to electronic records. EMR systems and other health information technologies rely heavily on critical users, particularly doctors. Therefore, the benefits of EMRs cannot be fully realized without user acceptance and approval. According to the literature review, effective criteria and subcriteria for evaluating attitudes toward the use of the OMOP system are listed in Table 1. The questionnaire used to assess attitudes toward the OMOP system included these components. Table 1 presents the criteria and subcriteria relevant to users’ attitudes toward the EMR system. The questions were designed using a 5-point Likert scale, ranging from 1=low to 5=high importance, allowing respondents to rate each subcriterion based on their perspective. The criteria include the ease of use, the usefulness of OMOP, the EMR system itself, and the quality of care, each with associated subcriteria reflecting the system’s usability, user-friendliness, and overall effectiveness.

**Table 1:** Effective criteria and subcriteria on the attitude of using OMOP^a^.

Effective criteria and subcriteria on the attitude of using OMOP		Code	Mean
**Ease of use [12,21,22,26]**			
	EMR^b^ resolution	E1	4.61
	EMR ease of use	E2	8.65
	Easy to remember EMR	E3	9.66
	User-friendliness of EMR	E4	6.64
	Getting started with EMR is easy	E5	5.61
**The usefulness of OMOP [12,21,22,26]**			
	EMR screen character resolution	U1	7.61
	Appropriateness and consistency of terms used in EMR	U2	5.57
	Appropriateness and consistency of the information used	U3	3.53
	Ease of learning the operation of the OMOP system	U4	9.57
	Features of the OMOP system	U5	5.52
**EMR systems [12,21,22,26]**			
	Using an EMR is a good idea	EM1	3.64
	Satisfaction with the use of EMR	EM2	1.65
	Does EMR save money?	EM3	6.34
	Does EMR save time?	EM4	1.68
	The use of EMR is useful for users	EM5	6.35
**Quality of care [12,21,22,26]**			
	OMOP quality	A1	4.62
	Usability of OMOP	A2	2.62
	Level of satisfaction with OMOP	A3	5.6
	The flexibility of the OMOP system	A4	2.59
	The power of the OMOP system	A5	7.59

^a^OMOP: Observational Medical Outcomes Partnership.

^b^EMR: electronic medical record.

## Methods

### Study Design and Overview

This methodology differs from previous studies in several key aspects. First, it significantly reduces the number of survey questions required to create a systematic causality diagram that decision makers can use. To thoroughly investigate the effectiveness of EMR systems and their relationship with attitudes toward the OMOP, a structured research methodology is essential. The research process is outlined in 3 key stages: The initial step involves establishing the relationship between EMR systems and users’ attitudes toward OMOP. FA will be applied to identify the underlying variables that influence users’ perceptions and satisfaction with OMOP within EMR systems. This method is crucial as it allows for the reduction of data complexity by identifying latent constructs that represent correlated variables. By understanding these correlations, we can better comprehend how different aspects of the EMR system influence users’ attitudes toward OMOP, thereby providing a clearer picture of user satisfaction and areas needing improvement.

The second stage involves determining the importance of various criteria and subcriteria related to EMR systems using the BWM. BWM is an MCDM approach that derives criteria weights efficiently by comparing the best and worst criteria against all others. This step is essential for prioritizing the factors identified in the first stage and understanding their relative importance in shaping user attitudes. By assigning precise weights, BWM quantifies the impact of each criterion, enabling targeted improvements and informed strategic decision-making. The final stage involves analyzing the interactions and dependencies among the criteria using the DEMATEL method. DEMATEL is a powerful tool for visualizing and understanding causal relationships among complex criteria. This method is crucial as it reveals how different criteria influence one another, providing insights into the systemic structure of the EMR system. Understanding these interdependencies is essential for identifying key leverage points and developing strategies to enhance overall system performance and user satisfaction. To summarize, the assessment framework is divided into 3 main stages:

- Determine the correlation between EMR systems and attitudes toward OMOP using FA.
- Determine the weight and importance of criteria and subcriteria using the BWM.
- Show how the criteria interact or depend on each other using the DEMATEL method.

The integration of BWM and DEMATEL results involves a systematic approach where the numerical outputs from each method inform and enhance the other. First, BWM is used to determine the relative importance (weights) of criteria and subcriteria by identifying the best and worst criteria through pairwise comparisons. These weights are then used as inputs for the DEMATEL method, which analyzes the interdependencies and causal relationships among the criteria. This combined approach allows for a comprehensive understanding of how different criteria influence each other and their overall impact on the system. By using the weighted criteria from BWM as a basis, DEMATEL provides a clearer understanding of how the most and least important criteria influence each other, refining the causal map of the system. The combined results offer a comprehensive view, where the weighted importance of criteria (from BWM) is contextualized within the network of influences and interactions (from DEMATEL). This integrated approach ensures that strategic decisions are both data driven and holistically informed. It allows for prioritizing factors not only based on their individual significance but also considering their dynamic interactions within the system.

DEMATEL is used to identify interrelationships and influences among criteria, revealing cause-effect chains, while BWM focuses on ranking and assigning precise weights to the criteria based on decision makers’ preferences for the best and worst options. For example, DEMATEL helps determine which criteria have the most significant influence on others, while BWM quantifies these influences into specific weights. The weights derived from BWM and the influence matrix from DEMATEL are combined using a weight aggregation method. This process involves normalizing the weights from both methods and integrating them to form a consolidated weight for each criterion. This approach ensures that both the hierarchical influence identified by DEMATEL and the precision of BWM are captured. Once the combined weights are obtained, they can be applied to MCDM models. For example, in a selection problem, the integrated model uses the combined weights to evaluate and rank alternatives, ensuring a balanced consideration of interrelationships and priority weights. The combined results must be validated against other decision-making methods and through performance analysis tests to ensure their reliability and applicability in real-world scenarios. This step involves comparing the outcomes of the integrated model with those from using DEMATEL or BWM alone, highlighting improvements in decision accuracy and robustness.

### Factor Analysis

FA is a statistical technique used to explore the latent structure of a data set. The primary goal of this study is to identify and examine the underlying factors that influence the observed variables. FA is commonly used in fields such as psychology, sociology, economics, and other social sciences [27].

FA is based on the principle that observed variables are influenced by a smaller set of latent factors. These latent factors are not directly observable but are inferred from patterns of correlations among variables. FA is a statistical technique that helps researchers understand the relationships between observed variables and the underlying factors that drive these relationships [25,28].

The BWM is an MCDM technique used to assess the relative weights or significance of selection criteria. This method involves selecting the best and worst criteria, with the best criteria representing those of greatest significance and the worst criteria representing those of least significance. In this study, BWM is used to determine the local weights for each criterion, as opposed to the analytic hierarchy process. Below, we provide a detailed explanation of the extended BWM calculation process [29-32].

### BWM Method

#### Introducing the Best-Worst Method: A More Efficient Approach to Pairwise Comparisons

This new method requires fewer pairwise comparisons compared with the analytic hierarchy process. In hierarchical analysis, the number of pairwise comparisons is given by the formula [*m* × (*m* – 1)]/2, where *m* is the number of criteria or indicators being compared. By contrast, the BWM reduces the number of pairwise comparisons to (2 × *m*) – 3, significantly decreasing the number of comparisons needed. The steps of this method are described in the following sections.

#### Step 1: Determining Research Criteria

In the first step, the decision matrix for the research problem is established, followed by the identification of the factors influencing the problem’s objective.

#### Step 2.1: Choosing the Set of Decision Criteria

During this step, it is essential to identify the most significant and least significant criteria from among all the indicators, termed as the best and worst criteria, respectively. Next, comparisons should be made between the best criteria and the other criteria, as well as between the other criteria and the worst criteria, using 2 separate matrices. Responses to these comparisons should be provided on a numerical scale ranging from 1 to 9. Overall, experts or decision makers establish evaluation criteria that align with the decision-making problem {*c*_1_, *c*_2_, …, *c_n_*}.

#### Step 2.2: Eliminating the Best and Worst Candidates

Once experts or decision makers have established the *q* standards in step 1, the best and worst criteria are selected. This step is crucial as it significantly impacts the analysis and outcomes.

#### Step 2.3: Creating the Best-to-Others Vector

Pairwise comparisons with other criteria should be performed using the best criterion [33]. The best-to-others vector is expressed as follows [34]:

*A_b_*=(*a_b_*_1_, *a_b_*_2_, ..., *a_bn_*) **(1)**

where the value of the best criterion *b*, which is superior to criterion *j*, is represented by *a_bj_*. By itself, the best criterion pairwise comparison must have a value of 1, or *a_bb_*=1.

#### Step 2.4: Creating the Other-to-Worst Vector

This step involves generating the other-to-worst vector using the worst criterion as a reference for comparisons with the worst [35]. The decision maker evaluates the remaining criteria on a scale of 1-9 relative to the worst criterion. The other-to-worst vector is then formulated, as shown in equation 2, based on these comparisons between the worst criterion and the other criteria.

*A_w_*=(*a*_1_*_w_*, *a*_2_*_w_*, …, *a_nw_*)*^T^*
**(2)**

where *a_jw_* denotes how much the remaining criterion *j* is more important than the least important criterion *w*. By itself, the worse criterion pairwise comparison must have a value of 1, *a_ww_*=1.

#### Step 3: Determining the Ideal Weight for Each Criterion (w*1, w*2, …, w*n)

During this stage, we construct the nonlinear optimization model of the BWM approach using the following equation [24,29]:



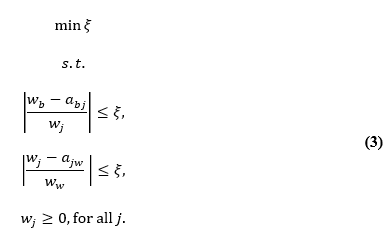



In this model, 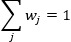.

When there are *n* criteria in total, the model can only compare pairs within a set of 4*n*–5 constraints in the solution of equation 6. Ultimately, the weights of all criteria combined are constrained to sum up to 1. In a nonempty collection, by assigning an appropriate value to ξ, a feasible solution space is created [29].

### DEMATEL

#### Understanding and Applying the DEMATEL Technique for Complex Problem Solving

The DEMATEL technique, developed between 1971 and 1976, is designed to address intricate and complex problems. Its purpose is to enhance the understanding of complex issues and interrelated problems, ultimately offering a clear solution through a hierarchical structure [17-20,24,29-44]. The DEMATEL technique involves the following key procedures. Its primary objective is to identify causal relationship patterns among a set of criteria. This method assesses the strength of communication through scoring, explores feedback and its significance, and recognizes relationships that are not easily transferable [24,36].

#### Step 1: Creating a Direct Relation Matrix

The first step is to create a direct relationship matrix. In this step, the effectiveness of each criterion is evaluated individually. When opinions from multiple people are used, their arithmetic mean is calculated. The dimensions of the evaluation scale are then established to accurately reflect the magnitude of the impact. Semantic operational definitions and values are categorized into a scale from 0 to 4, representing varying degrees of influence. The potential values are low impact (1), medium impact (2), high impact (3), and very high impact (4) [24].

Experts (evaluators) complete this questionnaire (matrix), rating the relative importance of pairs of criteria. The values specified in step 1 are used to generate the direct relationship matrix. A direct relationship matrix *A* = [*a_ij_*]_n__*__n_ is then created by integrating the responses from the different experts. The initial average matrix, denoted as *J*, is constructed by calculating the mean scores provided by the respondents. This process determines each element in the matrix *a_ij_*. The diagonal elements of the matrix are assigned a value of 0 [24].

#### Step 2: The Direct Influence Matrix Normalization

To normalize the direct correlation matrix, the formula *N*=*A*/*Z* (ie, equation 4) is used. To calculate *Z*, the sum of all rows and columns is first found. The largest resulting number is chosen as *Z*. Then, all the entries of the direct correlation matrix are divided by *Z*.



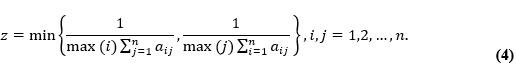



#### Step 3: Computing the T (Matrix of Total Influence)

To calculate the total correlation matrix *T* = [*t_ij_*]_n__*__n_, an identity matrix *I* is formed We then subtract the identity matrix from the normalized matrix that 
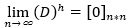
. Finally, we multiply the inverted matrix by the normal matrix. The total communication matrix is calculated from the relationship *T*=*N*×(*I* – *N*) – 1. In other words

*T* = *D* + *D*^2^ + ··· + *D^h^* = *D*(*I* – *D*)^–1^
**(5)**

#### Step 4: Drawing the Network Relation Map Relationships

Column vectors *R* and *S* represent the *T*’s column and row sums as equations 6 and 7 [24]:



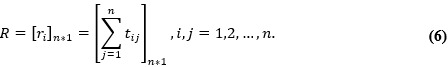





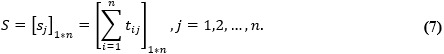



where [*S_j_*]*^T^*_1*__n_ = [*s_i_*]n*1. If *r_i_* represents the sum of matrix *T*’s *i*th row, then *r_i_* represents the total of factor *i’*s that affects every other factor. The total of the direct and indirect effects that factor *i* has gotten from all of the other factors is represented by *s_i_*, if *s_i_* represents the column sum from matrix *T*. Additionally, (*r_i_* + *s_i_*) provides an index of the strength of the effects that are imparted and received, meaning that (*r_i_* + *s_i_*) represents the extent of factor *i*’s overall effect in this system. The other factors are therefore influenced by factor *i* if (*r_i_* – *s_i_*) is positive; conversely, if (*r_i_* – *s_i_*) is negative, then factor *i* is generally influenced by the other factors [24,45,46].

#### Step 5: Creating a Causal Diagram

The summation of elements in each row (*D*) for every factor represents the impact of that factor on other elements within the system, reflecting the extent of influence exerted by the factor. Similarly, the summation of elements in each column (*R*) for each factor indicates the degree to which that factor is influenced by other elements in the system, showing the extent of influence received by the factor.

Consequently, the horizontal vector (*D* + *R*) represents the overall influence of the specific factor within the system. In simpler terms, a higher *D* + *R* value for a factor implies a greater level of interaction that the factor has with other factors in the system.

By contrast, the vertical vector (*D* – *R*) illustrates the influence of each factor. Generally, if *D* – *R* is positive, the factor is considered a causal variable; if it is negative, it is regarded as an effect.

#### Step 6: Drawing the Cartesian Coordinate Diagram

In conclusion, a Cartesian coordinate system is constructed where the longitudinal axis represents *D* + *R* values, and the transverse axis represents *D* – *R* values. Each factor’s position is defined by a point with coordinates (*D* + *R*, *D* – *R*) within this system. This approach results in a graphical diagram that visually represents the relationships and interactions among the factors in the system.

### Ethics Approval

This study did not involve human participants or animals, and therefore, no ethics approval was required.

## Implementation (Results)

### Demographic Information

In the first phase of our study, we performed an FA on data collected from 98 hospital visitors, all of whom are users of the AliHealth EMR system. The demographic details of these participants are presented in Table 2. In the second phase, we conducted an MCDM survey with 10 experienced physicians and specialists, each with over 10 years of experience and advanced degrees. They were knowledgeable about electronic health topics, EMR systems, and OMOP. Our research aimed to investigate the impact of the OMOP on AliHealth users. OMOP is a public-private initiative designed to enhance methods and tools for analyzing health care data, particularly for postmarket surveillance of medical products. Its primary goal is to develop a common data model that standardizes the structure and content of observational health data, facilitating more efficient and reliable analysis across different data sources [47]. This model facilitates the integration of various data sets, enabling researchers to perform large-scale analyses and generate evidence on the safety and effectiveness of medical products. By examining the role of OMOP within the EMR system, we aim to understand how this standardized model enhances data integration and analysis capabilities. Our findings will contribute to ongoing efforts to improve health care outcomes through better data management and analytical tools, ultimately benefiting both health care providers and patients by ensuring safer and more effective medical treatments. Descriptive statistical measures have been used to assess the demographic characteristics of the participants, as illustrated in Table 2.

**Table 2:** Demographic characteristics of the respondents (N=98).

Characteristics			Values
**Gender, n (%)**			
	Male	69 (70)	
	Female	29 (30)	
**Age (years), n (%)**			
	≤25	25 (26)	
	26-35	46 (47)	
	36-45	15 (15)	
	46-55	9 (9)	
	≥56	3 (3)	
**Marital status, n (%)**			
	Single	35 (36)	
	Married	63 (64)	
**Education, n (%)**			
	High school	17 (17)	
	Diploma	24 (24)	
	Bachelor	37 (38)	
	Masters	17 (17)	
	PhD	3 (3)	
**Use the online app, n/N (%)**			
	Everyday	18/55 (33)	
	2 or 3 times a week	29/89 (33)	
	Once a week	21/64 (33)	
	Once every 2 weeks	9/27 (33)	
	Once a month or less	21/64 (33)	
**See a doctor and update the medical record** **, n/N (%)**			
	Very little	10/30 (33)	
	Low	11/34 (32)	
	Medium	52/57 (91)	
	Much	14/43 (33)	
	Very much	12/37 (32)	

### FA Finding

**Figure 1:**
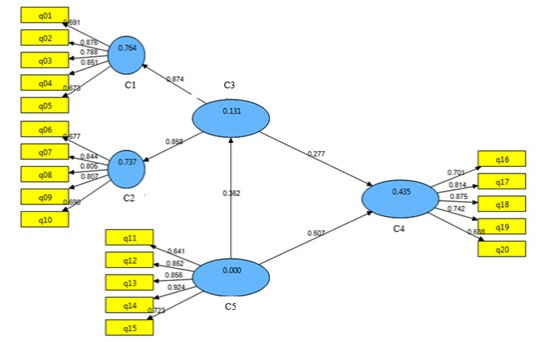
Constructing a comprehensive research model using the partial least squares.

**Figure 2:**
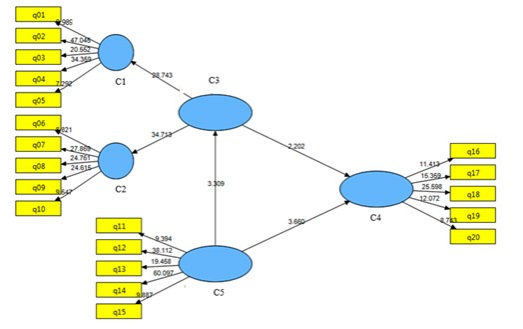
Utilizing the bootstrapping technique to derive T-statistics for the research model.

Data are considered unsuitable for FA if the Kaiser-Meyer-Olkin (KMO) value is below 0.5. When the KMO value ranges between 0.5 and 0.69, FA should be approached with caution. However, if the KMO score is above 0.7, the correlations in the data are deemed suitable for FA. In this research, a KMO score of 0.766 indicates that the data are appropriate for analysis. Figure 1 illustrates the results of the partial least squares analysis, while Figure 2 displays the *t* value.

The path coefficients and their significance are given in Table 3.

**Table 3:** Path coefficients.

*t* Value	Impact rate	Direction of the path
2.202	0.277	The effect of attitude toward the use of OMOP^a^ on quality of care
34.713	0.858	The effect of attitude toward the use of OMOP on the usefulness of OMOP
28.743	0.874	The effect of attitude toward the use of OMOP on ease of use of OMOP
3.66	0.507	The effect of EMR^b^ systems on quality of care
3.309	0.362	The effect of EMR systems on the attitude toward the use of OMOP

^a^OMOP: Observational Medical Outcomes Partnership.

^b^EMR: electronic medical record.

Table 3 reveals the strength of the relationships identified in the analysis. The correlation between EMR systems and attitudes toward OMOP usage is quantified at 0.362, with a test statistic of 3.309. This statistic exceeds the critical *t* value of 1.96 at the 5% significance level, confirming the statistical significance of the observed relationship. Therefore, with a 95% confidence level, a significant relationship between EMR systems and attitudes toward OMOP usage is established.

Similarly, the analysis reveals a relationship strength of 0.507 between EMR systems and quality of care, supported by a test statistic of 3.660. This result confirms a significant relationship between EMR systems and quality of care with 95% confidence.

Likewise, the correlation between the attitude toward OMOP usage and quality of care is 0.277, with a test statistic of 2.202. This statistic indicates a significant relationship between the attitude toward OMOP usage and quality of care with 95% confidence. Furthermore, the relationship strength between the attitude toward OMOP usage and the usefulness of OMOP is 0.858, with a test statistic of 34.713, which exceeds the critical *t* value at the 5% error level (1.96), confirming a significant relationship with 95% confidence. Therefore, a significant relationship is observed between the attitude toward OMOP usage and the usefulness of OMOP with 95% confidence. Additionally, the correlation between the attitude toward OMOP usage and the ease of use of OMOP is quantified at 0.874, with a test statistic of 28.743. This confirms a significant relationship between the attitude toward OMOP usage and the ease of use of OMOP with 95% confidence.

### DEMATEL Findings

As outlined in the paper, the research questionnaire was developed using the DEMATEL technique and then administered to the participants. Table 4 presents the average opinions of the experts regarding the impact of each criterion (rows) on the other criteria (columns).

We use the formula 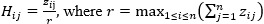 to normalize the components of Table 5.

**Table 4:** Average opinion of all experts.

Opinion	C1	C2	C3	C4	C5
C1	1.492	0.72	0.725	0.738	0.648
C2	0.867	1.707	0.96	1	0.779
C3	0.475	0.571	1.47	0.711	0.454
C4	0.693	0.825	0.872	1.723	0.852
C5	0.639	0.712	0.672	0.746	1.493

Table 5 shows the matrix after normalization.

**Table 5:** Normalized matrix.

Matrix	C1	C2	C3	C4	C5
C1	0.182	0.152	0.182	0.213	0
C2	0.152	0.273	0.273	0	0.303
C3	0.03	0.273	0	0.152	0.091
C4	0.333	0	0.242	0.214	0.121
C5	0	0.182	0.121	0.215	0.182

After computing the matrices mentioned above, the total relations matrix is derived using the following formula: 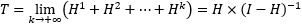


In this formula, I is the unity matrix. The calculation results of the T matrix are shown in Table 6.

**Table 6:** The total relationship matrix (T).

Matrix	C1	C2	C3	C4	C5
C1	0.648	0.738	0.725	0.72	0.492
C2	0.779	1	0.96	0.707	0.867
C3	0.454	0.711	0.47	0.571	0.475
C4	0.852	0.723	0.872	0.825	0.693
C5	0.493	0.746	0.672	0.712	0.639

The next step is to obtain the sum of the rows and columns of the matrix *T*. We obtain the sum of rows and columns according to the following formula: 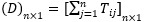 and 
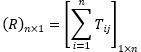
.

In the provided equation, *D* and *R* represent matrices of dimensions *n*×1 and 1×*n*, respectively. The next phase is evaluating the importance of indicators (*D_i_* + *R_i_*) and the correlation between criteria (*D_i_* – *R_i_*). If the difference between *D_i_* and *R_i_* is more than 0, the corresponding criterion is regarded as effective; conversely, if the difference between *D_i_* and *R_i_* is less than zero, the corresponding criterion is judged effective.

**Figure 3:**
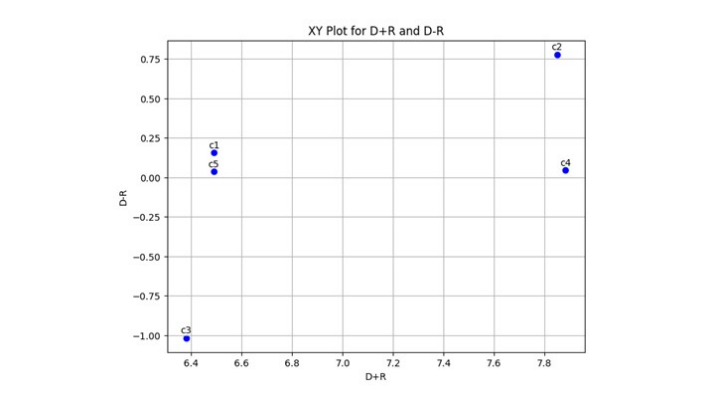
XY plot for D+R and D-R.

**Figure 4:**
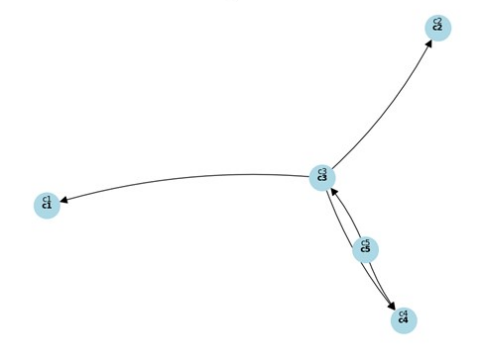
XY plot for importance and influence.

Table 7 and Figure 3 show the *D_i_* + *R_i_* and *D_i_* – *R_i_* and *XY* plots for importance, with their influence shown in Figure 4.

**Table 7:** Obtaining the importance and influence of criteria.

Matrix	Criteria	*D*	*D*_*i*_ + *R*_*i*_	*D*_*i*_ – *R*_*i*_
C1	The usefulness of OMOP^a^	3.324	6.49	0.157
C2	Ease of use of OMOP	4.314	7.849	0.778
C3	Attitude toward the use of OMOP	2.681	6.38	-1.018
C4	Quality of care	3.964	7.883	0.046
C5	Electronic medical record systems	3.262	6.489	0.036

^a^OMOP: Observational Medical Outcomes Partnership.

### BWM Finding

The BWM is an effective MCDM technique used to determine the weights of criteria. The process begins by selecting the best and worst criteria based on expert judgment or specific research objectives. In this study, “The usefulness of OMOP (C1)” is identified as the best criterion, and “Electronic Medical Record Systems (C5)” is identified as the worst criterion. Following this, pairwise comparisons are performed between these criteria and all other criteria. The next step involves constructing a pairwise comparison matrix where comparisons are made between the best criterion (C1) and the other criteria, as well as between the worst criterion (C5) and the remaining criteria. Experts assign values based on how much better or worse one criterion is compared with another. An optimization model is then developed to minimize the maximum absolute differences between the derived weights and the optimal consistency ratio, ultimately determining the weights of the criteria. The weights assigned to the criteria in this analysis are as follows: “Usefulness of OMOP (C1)” has a weight of 0.3971, “Ease of use of OMOP (C2)” has a weight of 0.1985, “Attitude toward the use of OMOP (C3)” has a weight of 0.1489, “Quality of care (C4)” has a weight of 0.1538, and “Electronic medical record systems (C5)” has a weight of 0.1017. These weights reflect the relative importance of each criterion in the context of the research. The detailed results of the BWM analysis are presented in Table 8.

Table 8 shows the weights assigned to various factors related to the OMOP framework. The weights are as follows: W1 for “Usefulness of OMOP,” W2 for “Ease of use of OMOP,” W3 for “Attitude toward the use of OMOP,” W4 for “Quality of care,” and W5 for “Electronic medical record systems.” Analysis of these weights reveals that W1 (Usefulness of OMOP) has the highest weight of 0.3971, indicating that this factor is considered the most important in the evaluation. By contrast, W5 (Electronic medical record systems) has the lowest weight of 0.1017, suggesting that it is considered less critical in this context. The weights provide a quantitative measure of the relative importance of each factor, with a higher weight indicating greater significance in the overall assessment of the OMOP framework. The process begins by selecting the best and worst criteria based on their relative importance, as reflected in their weights. After identifying these criteria, pairwise comparisons are conducted between the best and worst criteria and all other criteria. Following the pairwise comparisons, a linear programming model is formulated and solved to minimize the maximum deviation between the derived and optimal consistency ratios. Expert evaluations were aggregated using the simple weighted average method. The weights determined by this process are presented in Table 8.

**Table 8:** BWM_a_ result for subcriteria weight.

Criteria and subcriteria		Values
**The usefulness of OMOP^b^**		0.39708431
	U1	0.31007783
	U2	0.1860467
	U3	0.23255837
	U4	0.15503891
	U5	0.11627919
Attitude toward the use of OMOP		0.14890674
**Ease of use of OMOP**		0.19854216
	E1	0.19460294
	E2	0.19041054
	E3	0.12694036
	E4	0.10722507
	E5	0.38082109
**Quality of care**		0.15375285
	A1	0.38793627
	A2	0.19707721
	A3	0.12694036
	A4	0.10722507
	A5	0.18082109
**Electronic medical record systems**		0.10171394
	EM1	0.29528318
	EM2	0.07827498
	EM3	0.17854757
	EM4	0.24996659
	EM5	0.19792768

^a^BWM: best-worst method.

^b^OMOP: Observational Medical Outcomes Partnership.

These weights are shown in Multimedia Appendices 1 and 2 (the subcriteria weight is per Multimedia Appendix 2).

Table 8, along with Multimedia Appendices 1 and 2, assigns weights to various components related to the usefulness of OMOP. Among these components, “EMR resolution” has the highest weight, indicating its significant role in the evaluation. Conversely, “EMR ease of use” has the lowest weight, suggesting it is considered less critical in this context. The specific weight values are as follows: “EMR resolution” is assigned a weight of 0.31007783, while “EMR ease of use” has a weight of 0.1860467. This suggests that, in the assessment of EMR systems, stakeholders place the highest importance on the resolution aspect, while comparatively less weight is given to the ease of use of the EMR system. For the ease of use of OMOP, “EMR screen character resolution” holds the highest weight, highlighting its substantial importance in the assessment. Conversely, “Features of the OMOP system,” “Appropriateness and consistency of the information used,” and “Ease of learning the operation of the OMOP system” share the lowest weight, indicating a relatively lower significance for these aspects. The specific weight values are as follows: “EMR screen character resolution” has a weight of 0.19460294, while “Features of the OMOP system,” “Appropriateness and consistency of terms used in EMR,” and “Ease of learning the operation of the OMOP system” each have a weight of 0.12694036. This indicates that, in evaluating the EMR system within the OMOP framework, stakeholders consider the clarity and quality of screen character resolution to be the most important factor. By contrast, the features, terminology, and ease of learning are viewed as less influential.

For “Quality of care,” the component “Using an EMR is a good idea” has the highest weight, indicating that stakeholders place significant importance on the overall concept of using an EMR system. “Satisfaction with the use of EMR” follows closely, suggesting that user satisfaction is also a key consideration in the evaluation. By contrast, “Does EMR save time?” has the lowest weight, implying that the time-saving aspect is considered less critical in this context. The specific weight values are as follows: “Using an EMR is a good idea” is assigned a weight of 0.38793627, indicating the highest priority. “Satisfaction with the use of EMR” has a weight of 0.19707721, reflecting its importance in stakeholder evaluations. “The use of EMR is useful for users” is assigned a weight of 0.18082109. By contrast, “Does EMR save money?” has a weight of 0.12694036, and “Does EMR save time?” has a weight of 0.10722507, suggesting that considerations related to time and cost savings are less emphasized compared with the perceived value and user satisfaction associated with EMR.

For “Electronic medical record systems,” the component “OMOP quality” has the highest weight, reflecting the significant importance stakeholders place on the overall quality of the OMOP system. “The power of the OMOP system” is the next highest, indicating that the system’s capability and strength are also crucial factors. Conversely, “Usability of OMOP,” “Level of satisfaction with OMOP,” and “The flexibility of the OMOP system” all share lower weights. This suggests that while usability, user satisfaction, and system flexibility are relevant, they are considered less critical compared with overall quality and system power in this context. The specific weight values are as follows: “OMOP quality” has a weight of 0.29528318, indicating a high priority for the overall quality of the OMOP system. “The power of the OMOP system” is assigned a weight of 0.19792768, reflecting its significant importance in the evaluation. By contrast, “Usability of OMOP,” “Level of satisfaction with OMOP,” and “The flexibility of the OMOP system” each have a weight of 0.07827498. This distribution suggests that stakeholders place greater emphasis on the overall quality and power of the OMOP system, while usability, satisfaction, and flexibility are considered relatively less critical in the evaluation process.

## Discussion

### Principal Findings

The primary objective of this research was to evaluate key components related to the usefulness, ease of use, quality of care, and EMR systems within the OMOP framework. The study aimed to identify the most critical factors influencing stakeholders’ perceptions and priorities in these areas. The results, presented in Table 8 and Multimedia Appendices 1 and 2, highlight that “EMR resolution” and “OMOP quality” received the highest weights, indicating their significant importance in the assessment. Conversely, aspects such as “EMR ease of use” and “flexibility of the OMOP system” were considered less critical. Information technology plays a crucial role in enhancing data quality, which in turn improves patient care and safety. It is important for experts to define and explain the characteristics of health data quality so that system designers can identify appropriate methods to evaluate and improve these aspects during system revisions. This will contribute to better information quality in predictive and embedded systems. The findings of this study, particularly the emphasis on “EMR resolution” and “OMOP quality” as critical factors in evaluating the usefulness, ease of use, and quality of care within the OMOP framework, align well with existing literature. The focus on technical quality and the significance of detailed, high-resolution data for effective EMR use are consistently supported by multiple studies. For instance, the study by Kohane et al [48] on the impact of EMR systems on primary care practices underscores the importance of structural and process-related benefits that depend on high-resolution and quality EMR data to improve clinical outcomes. Additionally, the study by Lánczky and Győrffy [49] highlighted the usability of EMRs among health care professionals across various sectors, emphasizing the critical role of technical quality in facilitating efficient clinical workflows and decision-making processes. Effective EMR implementation in mental health settings depends on usability, acceptance, and alignment with clinical needs and workflows, although long-term outcomes remain unclear. Moreover, our study’s conclusion that “EMR ease of use” and “flexibility of the OMOP system” were deemed less critical aligns with broader literature, which often underscores that user satisfaction is more strongly influenced by the quality and resolution of EMR data than by its ease of use. For example, a study [50] assessing the implementation of EMRs in mental health settings found that while ease of use is important, the perceived quality and accuracy of the data captured by the system were more significant in influencing user satisfaction and overall system acceptance.

The results related to the user interface reveal that users rated the clarity and quality of the EMR system screen, the appropriateness and consistency of the terms and information used, and the ease of learning its functions and capabilities as crucial factors. This indicates that, during the design and implementation of the EMR system, considerable attention has been given to external factors influencing system acceptance. When designing a new system, it is important to consider not only its functionality but also its appearance and all aspects that impact user interaction and satisfaction. Previous studies have found that more than 96.6% of doctors and midwives believe the EMR system is easy to operate, learn, and use [38-42]. In our study, the average user score for the ease of use of the system was 64.25. Regarding user attitudes toward EMR usage, polyclinic users exhibited a positive attitude with an average score of 66.84. This score is notably higher than the score (55.75) reported previously, indicating a favorable disposition toward the EMR system among polyclinic users. This positive attitude suggests that, with attention to other factors, there is potential to further enhance user satisfaction with the EMR system. In our study, the average behavioral preference score for the EMR system is 61.39. This score reflects users’ preferences for the system, including its attractiveness, excitement, flexibility in adapting to changes, and overall power. These factors are related to users’ perceptions of the system and are influenced by external factors associated with its implementation and use.

### Conclusions

Based on the average scores for external factors such as data quality, clarity, and the user interface, it is evident that significant attention was given to these aspects during the design of the EMR system to ensure user satisfaction. Most users view the use of OMOP within the EMR system as beneficial, noting that this technology enhances productivity and fosters a positive attitude by improving task performance. This improvement can be attributed to reducing task completion times and providing timely information. Over half of the EMR system users find it easy to use, believing that learning to operate the system requires minimal mental effort. Most users hold a positive attitude toward the EMR system. Consistent with these positive attitudes, the behavioral tendency to use the EMR system is also favorable. Future research should consider using the proposed integrated model to assess potential obstacles in the EMR system.

## Appendix

Multimedia Appendix 1 Best-worst method result for criteria weight.

Multimedia Appendix 2 Best-worst method result for subcriteria weight.

## Abbreviations

BWM: best-worst method
DEMATEL: Decision-Making Trial and Evaluation Laboratory
EHR: electronic health record
EMR: electronic medical record
FA: factor analysis
KMO: Kaiser-Meyer-Olkin
MCDM: multicriteria decision-making
OMOP: Observational Medical Outcomes Partnership
VIKOR: Vlsekriterijumska Optimizacija i Kompromisno Resenje

## Data Availability

Data for this study are available from the corresponding author upon reasonable request.
