# Supplementary figures and images for: Implementation of the Observational Medical Outcomes Partnership Model in Electronic Medical Record Systems: Evaluation Study Using Factor Analysis and Decision-Making Trial and Evaluation Laboratory-Best-Worst Methods
Source: JMIR Med Inform. 2024 Sep 27;12:e58498. doi: 10.2196/58498 (PMC11470222; doi:10.2196/58498)

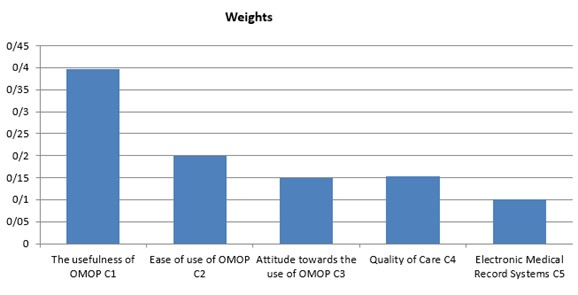

Supplement: Multimedia Appendix 1 [file medinform_v12i1e58498_app1.png]

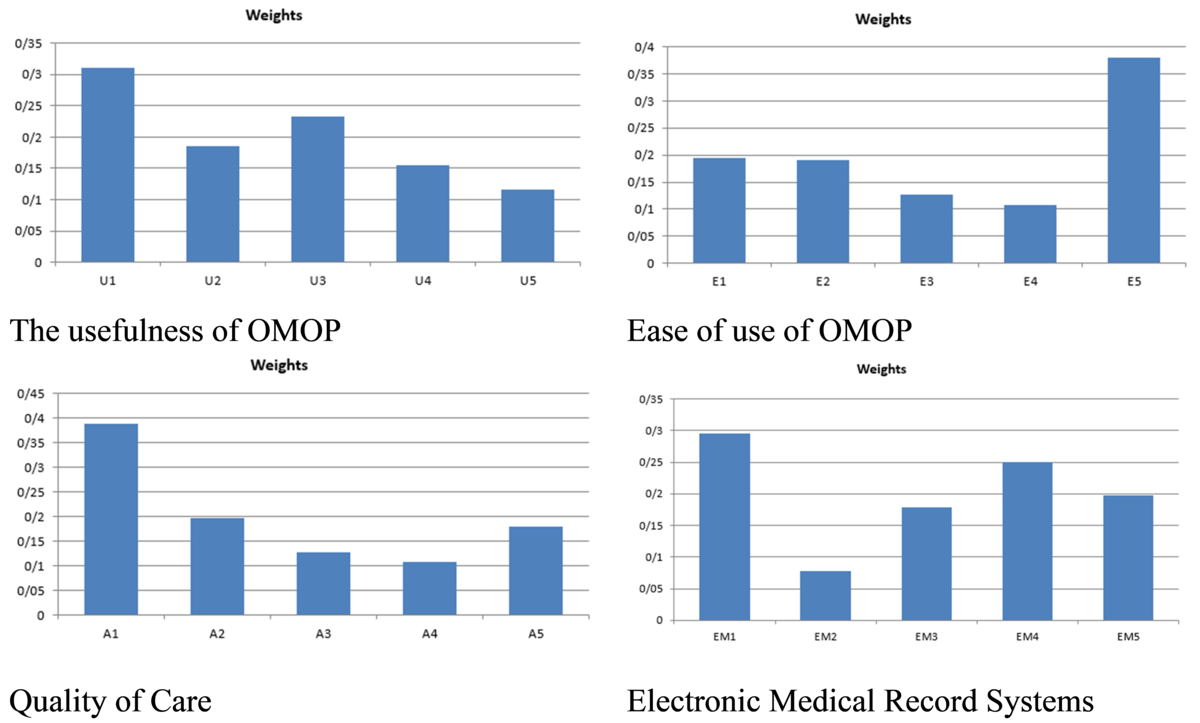

Supplement: Multimedia Appendix 2 [file medinform_v12i1e58498_app2.png]
